# A Customized Knee Antibiotic-Loaded PMMA Spacer: A Preliminary Design Analysis

**DOI:** 10.3390/polym13234065

**Published:** 2021-11-23

**Authors:** Marco Balato, Carlo Petrarca, Antonio Quercia, Aniello Riccio, Andrea Sellitto, Jessica Campi, Anna Borriello, Mauro Zarrelli, Giovanni Balato

**Affiliations:** 1 Department of Electrical Engineering and Information Technologies, University of Naples “Federico II”, 80125 Napoli, NA, Italy; carlo.petrarca@unina.it (C.P.); antonio.quercia@unina.it (A.Q.); 2 Department of Engineering, University of Campania “Luigi Vanvitelli”, 81031 Aversa, CE, Italy; aniello.riccio@unicampania.it (A.R.); andrea.sellitto@unicampania.it (A.S.); jessica_campi@hotmail.it (J.C.); 3 National Research Council of Italy (CNR), Institute for Polymers, Composite and Biomedical Materials (IPCB), 80055 Portici, NA, Italy; anna.borriello@cnr.it (A.B.); mauro.zarrelli@cnr.it (M.Z.); 4 Department of Public Health, University of Naples “Federico II”, 80131 Naples, NA, Italy; giovanni.balato@unina.it

**Keywords:** spacer, 3D printing, experimental analysis, FEM simulations

## Abstract

A preliminary design of customized antibiotic-loaded poly-methyl-methacrylate (ALPMMA) spacer characterized by an appropriate footprint according to the specific patient’s anatomy and a reliable mechanical response to severe functional loads (i.e., level walking and 45° bent knee) is reported. The targeted virtual prototyping process takes origin from a novel patented 3D geometrical conceptualization characterized by added customization features and it is validated by a preliminary FEM-based analysis. Mechanical and thermomechanical properties of the antibiotic-doped orthopedic PMMA cement, which will be used for the future prototype manufacturing, were measured experimentally by testing samples taken during a real day-running orthopedic surgery and manufactured according to the surgeon protocol. FEM analysis results indicate that small area is subjected to intensive stresses, validating the proposed geometry from the mechanical point of view, under the two loading scenarios, moreover the value of safety margins results positive, and this is representative of the lower stress magnitude compared to the critical material limits. The experimental data confirm that the presence of antibiotic will last during the surgeon period moreover, the temperature dependent modulus of the bone cement is slightly affected by the body range temperature whereas it will drastically drop for higher temperature out the range of interest. A complete customization, according to a patient anatomy, and the corresponding real prototype spacer will be manufactured by 3D printing techniques, and it will be validated by destructive testing during the second stage of this activity before commercialization.

## 1. Introduction

Joint replacement (JR) is a life-improving procedure affecting millions of people worldwide each year. Successful JR significantly affects the patients’ personal autonomy and quality of life. Although JR is already a frequently performed procedure, the incidence rate of prosthesis implantation is expected to continue to rise [1,2,3,4,5]. It is estimated that in the United States (US), in 2040, the number of primary total hip and total knee replacement (THR, TKR) will increase of 284% and 401%, respectively [5]. Despite the majority of JR operations offering pain relief, and potential functional and quality of life improvement, a minority of patients will require additional surgery as a result of both aseptic and septic failures [6,7,8]. The aseptic failure, affecting the 28% of the total JRs, includes loosening at the bone cement interface, periprosthetic fracture, fracture, wear, implant malposition, dislocation-instability, or materials fatigue [8]. Prosthetic joint infection (PJI) is defined as one of the most devastating complications in the field of orthopedic surgery. Although the improvements of implant materials, surgical technique and the administration of antibiotic prophylaxis, the rate of PJI has been increasing [8,9]. In US, the number of hip revisions, between 2005 and 2030, is estimated to rise by 137%, with a national total cost of USD 753.4 million [10,11,12,13]. Furthermore, the estimated national total hospital cost for infected TKR is projected to be USD 1.1 billion annually. In the United Kingdom (UK), instead, between 2012 and 2030, demand for hip revision was projected to rise by 31% [14]. At the hospital level, research has found that reimbursement for revision arthroplasty for PJI does not meet the cost, suggesting an increased financial burden on treating hospitals [15,16]. Currently, once the local septic failure occurs, the two-stage technique with the use of an antimicrobial impregnated polymethylmethacrylate (PMMA) spacer represents the best treatment option with an infection eradication rate that ranges from 85% to 100% [17,18,19]. Two stage technique consists of two surgical procedures. At time of the first surgical stage, joint prosthetic implant is removed, and an accurate debridement of all infected tissue is performed. Subsequently, an antibiotic loaded cement spacer is inserted in joint space. The second surgical stage, performed after clinical and laboratory have normalized, provides an additional debridement of the joint space and the implant of revision joint prosthesis after spacer removal [20].

Antibiotic-loaded PMMA (ALPMMA) spacers aims to preserve anatomical structures, and joint mobility and to allow infection cure by releasing antibiotics locally. Moreover, ALPMMA spacer favor the re-implantation of the definitive prosthesis during the second stage of PJI treatment [20,21,22,23,24,25,26]. Typically, mobile spacers have the same geometric shape of a primary prosthetic implant and may be either preformed or directly prepared in the operating room using plastic molds. ALPMMA spacers, currently available on the market, show a series of disadvantages, closely linked to a low propensity to customize, seen as the ability to adapt to the patients’ anatomical characteristics, with consequential increase in surgical complexity, surgery duration and post-operative complications.

Conventionally, ALPMMA spacers are available only in three or four standard sizes, with the impossibility to guarantee the perfect matching of ALPMMA spacers with residual bone (no further bone loss) and gap filling. For these reasons, to obtain a well clinical and functional results, the surgical procedure necessitates an increased time than joint prosthesis implantation. The surgical time is correlated to patient’s blood loss [26] and increase in post-operative complications such as surgical site wound problems and thromboembolic events. Furthermore, PMMA spacers are correlated to a higher risk of fractures and dislocation. The addition of antibiotic to PMMA reduces the mechanical properties of bone cement thus causing breakage.

No studies have been conducted to introduce design guidelines of ALPMMA spacers as a tool to combine the geometric characteristics of the spacers with its mechanical and functional properties. 

The present paper aims to evaluate the effect of possible customization on the mechanical performance in operating and simulated worst loading conditions. The expected result is a customized ALPMMA spacer that shows not only an appropriate footprint, as a function of patient’s anatomical behavior but also an adequate response to mechanical stresses. The paper is organized as follows: the proposed ALPMMA geometric 3D model is presented describing the customization feature of the model, which is later used for the FEM numerical simulations. 

The spacer material is characterized to acquire the necessary information regarding the mechanical properties according to medical standard and procedure, ultimately conclusions are drawn to pave the way for future optimization studies toward the complete customization according to the patients.

## 2. Material and Methods

### 2.1. Theoretical Background

**Figure 1:**
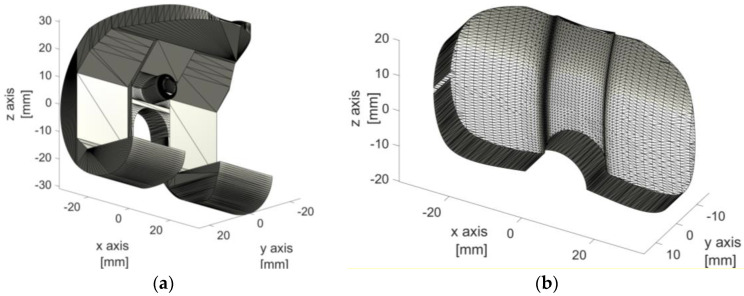
3D view of the proposed ALPMMA: (**a**) femoral and (**b**) tibial component.

Standard ALPMMA spacers are often used during staged revision arthroplasty due to bio-film-related infection in cases of extensive or asymmetrical bone loss since standard spacers may not preserve alignment, stability, and anatomical matching, thus causing failure of the procedure. In the following, a not exhaustive list of drawbacks associated with the common used ALPMMA spacers are reported, such as: (I) both femoral-tibial and femoral-patellar instability; (II) the possibility of developing important contractures in flexion; (III) frailty and subsequent breaking of the implant, especially with high doses of antibiotic; (IV) the difficulty of managing malalignments on the three planes of space (frontal, sagittal, and transversal) and bone defects whether segmental or cavitary (affecting articular surfaces, metaphysis, and diaphysis). The aforementioned weaknesses may be overcome through the designing of a patient-tailored ALPMMA spacer with specific features to mitigate the above mentioned drawbacks [27]. The objective is to introduce the additive manufacturing approach in the orthopedic field as a powerful tool to effectively manage the PJI. In the customization process, which occurs in two consecutive phases, such as 3D surgery planning phase and 3D printing, the starting point is represented by the identification of ALPMMA 3D spacer model. The proposed 3D model has been designed using Simulink 3D Animation Toolbox environment and it consists of femoral (Figure 1a) and tibial (Figure 1b) components resembling the geometry of a permanent artificial knee joint. In particular, Figure 1 refers to a proposed ALPMMA 3D spacer model in its standard size.

**Figure 2:**
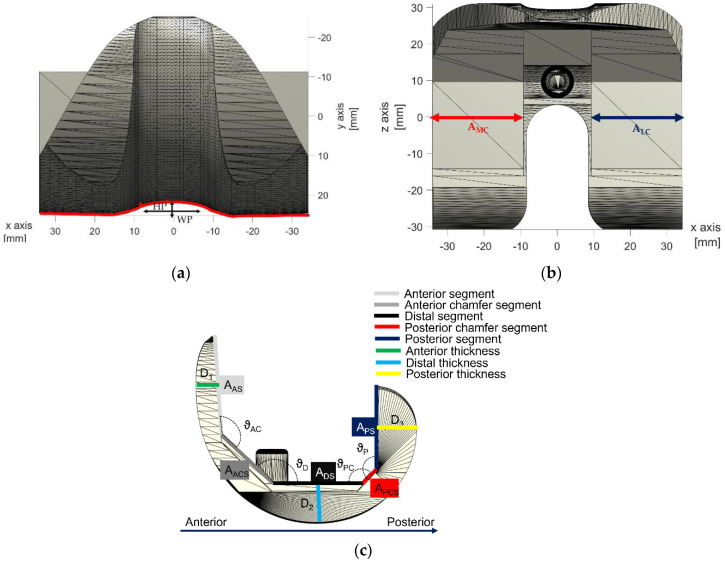
3D view of the proposed ALPMMA femoral component: (**a**) Anterior view, (**b**) top view and (**c**) A-P view.

To evaluate the cause-and-effect link between the geometric characteristics of the ALPMMA spacer along with the surgery and mechanical failures, a multivariable oriented design was set and implemented. The result is an ALPMMA spacer configuration with different degrees of freedom in terms of geometrical parameters to act with to manage efficiently according to the actual patient the alignment, the stability and the anatomical matching (see Figure 2).

Femoral condyles were shaped on both sides to effectively reduce pressure on the soft tissue and on anterior patellar. Moreover, to improve sliding and thus reducing the risk of adverse reactions, such as dislocation and ringing after surgery, it is possible to customize the shape of the anterior condyle (red curve in Figure 2a) by acting on the parameters HP and WP that affect its thickness and amplitude, respectively, allowing the patellar to enter the carriage movement earlier. Additionally, the proposed 3D model allows to carefully plan the encumbrance of the femoral component both in Antero-Posterior (A-P) and Medio-Lateral (M-L) size. The objective is to achieve an ALPMMA spacer with an arbitrary ratio between the A-P and M-L size as effective tool to correct the potential axial deformations assuring a proper fixation and at the same time, to manage bone defects properly.

**Figure 3:**
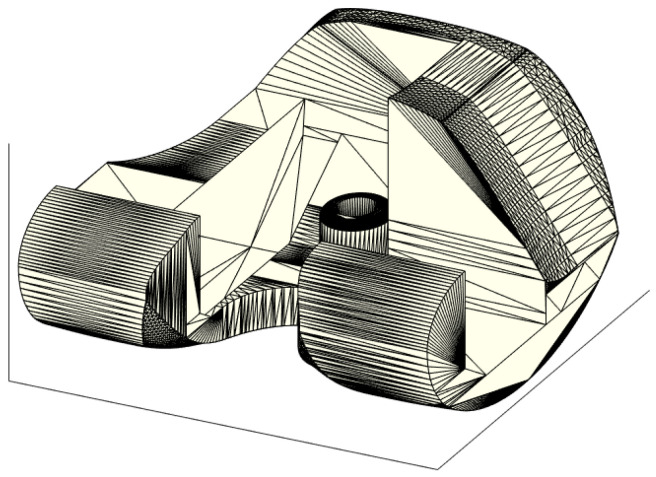
3D view of the proposed augmented ALPMMA femoral component.

From Figure 2b,c, it is evident how the footprint of the ALPMMA femoral spacer could be modified by editing the thickness (e.g., D_1_, D_2_ and D_3_), the amplitude of the two femoral internal condyles (e.g., A_MC_ and A_LC_) and also the amplitude of cutting segments (e.g., A_AS_, A_ACS_, A_DS_, A_PCS_, and A_PS_) along with corresponding angles (e.g., ϑ_AC_, ϑ_D_, ϑ_PC_, and ϑ_P_). Finally, an augmented ALPMMA femoral spacer is expected to correct severe bone defects reducing after implantation pain and time of recovery. In Figure 3, an example of asymmetric augmented ALPMMA femoral spacer is reported.

**Figure 4:**
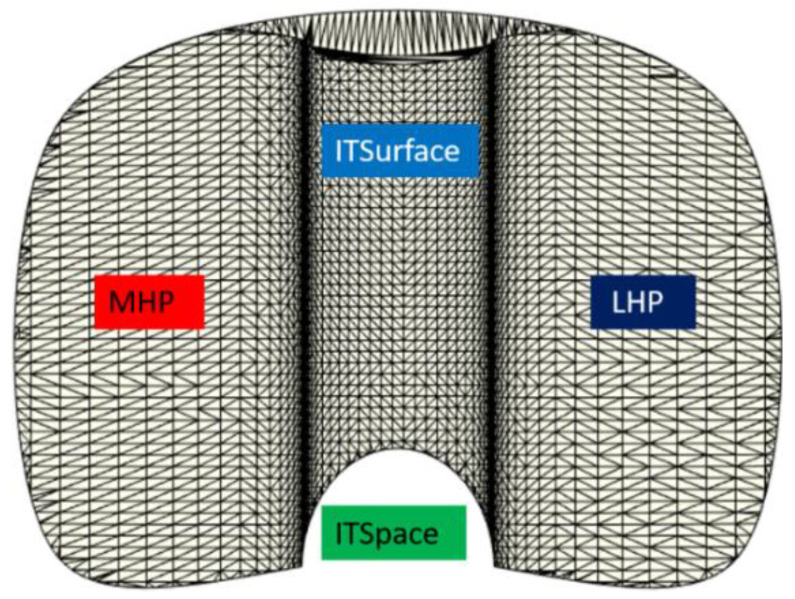
Top view of the proposed ALPMMA tibial component.

The proposed 3D model provides a multitude of degrees of freedom also for the tibial component. In fact, as shown in Figure 4, the 3D tibial model comprises three different portions: two Hemi-Plateaus (MHP and LHP) divided by an Intercondylar Tibial Space (ITSpace) and an Inter-condylar Tibial Surface (ITSurface). Geometrical characteristics, meant as width and length of both condylar portions are fully editable, and they are completely independent one from an another. This ALPMMA tibial spacer geometry results asymmetric and thus more suitable to shape the specific patient’s anatomy leading to a full customization in terms of geometry for better medical treatment and subsequent convalesce. The result is an asymmetric ALPMMA tibial spacer that reflects the patient’s anatomy allowing so it is better positioning on the transversal plane. At the end, also for the tibial component, it is possible to obtain an augmented ALPMMA tibial spacer to correct severe bone defects.

### 2.2. Experimental Characterization

**Figure 5:**
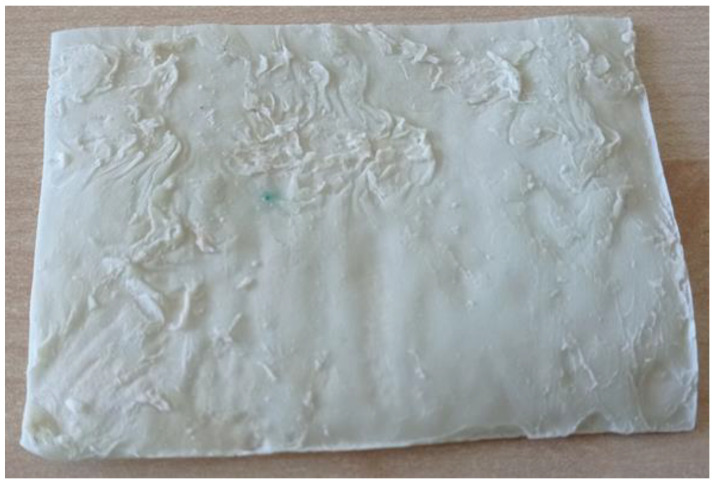
Hand mixed antibiotic cement taken from day surgery activity.

In order to correctly and accurately predict the mechanical behavior of the proposed geometry, experimental tests are mandatory to retrieve the material properties of the bone cement. The material considered for the prototype of the proposed spacer is a polymethylmethacrylate system loaded with 20% by weight of zirconium dioxide, kindly provided by Zimmer-Biomet Ltd. (Warsaw, IN, USA) [28]. The antibiotics were mixed by hand with PMMA copolymer powder at specific percentage and later added to the liquid monomer to attain the requested antibiotic bone cement (Figure 5). All the tested specimens were manufactured by casting the material supplied by the surgeon and prepared during daily orthopedic surgery activity and thus strongly dependent by the acting medical operator. To increase the antimicrobial spectrum, two antibiotics have been chosen, such as vancomycin and gentamicin. Two hundred and forty mg of gentamicin (6 mL) (Fisiopharma, Salerno, Italy) was added to 20 mL of liquid monomer and subsequently mixed with 40 g of cement powder. Two grams of vancomycin hydrochloride powder (Fisiopharma, Italy) was added following thirty seconds of mixing by hand the cement powder with liquid monomer with gentamicin [21].

Indeed, literature data on the mechanics of bone cements could not be directly compared, due to the induced variability associated with the preparation, the storage of sample and also testing conditions. Hence, in this work, compressive and flexural strengths have been measured experimentally following the recommendation of the ASTM standards, respectively, F451, D5023, and E831 and laboratory protocol for thermos-gravimetrical measurements, respectively. These tests were carried out to evaluate, respectively: the compressive modulus, the temperature dependency of the flexural modulus, the mass loss and the coefficient of thermal expansion. More in details, the compression modulus has been measured according to the indication of ASTM F451 on cylindrical shaped specimens; the effect of the temperature on flexural modulus was determined by Dynamic Mechanical Analysis according to the ASTM D5023, under three point bending configuration under force ramp mode. by using a 1 Hz frequency value and an elastic displacement of 40 μm with a temperature rate of 1 °C/min. In addition, a thermogravimetric analysis (TGA) was carried out, to verify the mass loss as a function of time, at constant temperature (i.e., internal body temperature of 37 °C), which can be related to the antibiotic released in the human body when the spacer is in position and during the normal service period. Finally, a characterization test was carried out to evaluate the thermal expansion co-efficient of the material in three different temperature ranges.

## 3. Result and Discussion

### 3.1. Compressive Tests

**Figure 6:**
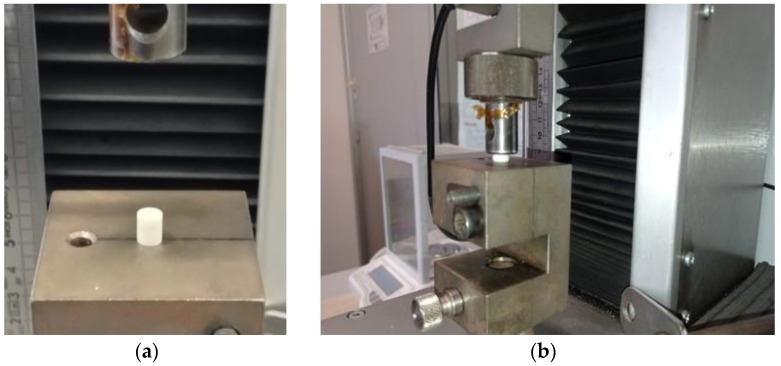
Compression test: (**a**) initial sample positioning; (**b**) compressed sample during the test.

The compressive tests have been carried out according to the standard ASTM F451 on cylinder shaped specimens with nominal dimension of 12 mm high and 6 mm in diameter (see Figure 6). For statistical purpose, five different tests were performed taking the set of samples from the same surgeon supplied mixture.

**Figure 7:**
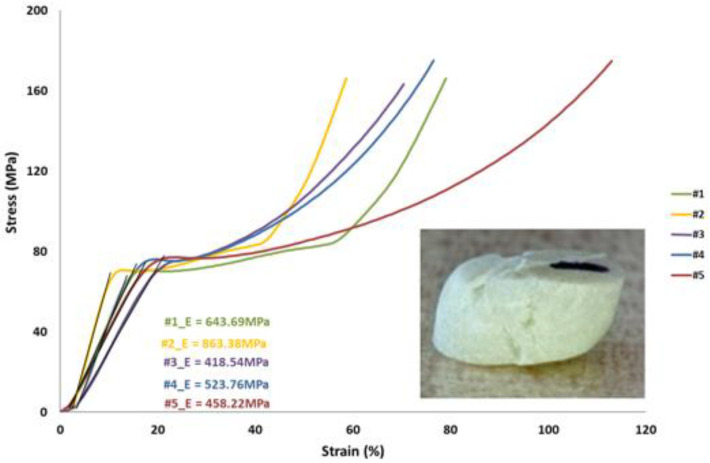
Compression test results.

The compression stress vs. strain curves are shown in Figure 7. Although the manufacturing process of the sample is likely affected by a spread variability due to the contingency of the surgery event by the medical operator, the curves reveal an appreciable repeatability with an average modulus of elasticity of ~582 MPa and a failure strength of ~175 MPa. 

### 3.2. Dynamic Mechanical Analysis

**Figure 8:**
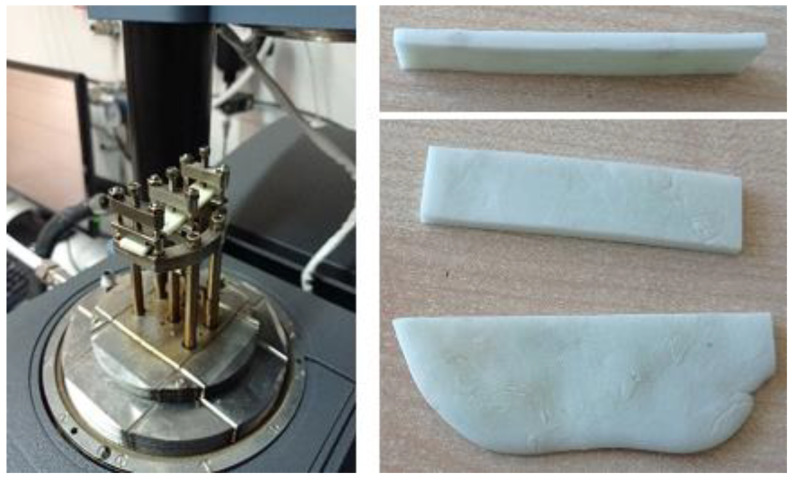
Three-point bending test.

The three-point bending tests have been performed according to the ASTM D5023 (see Figure 8). This test procedure is a standard protocol for measuring the dynamic mechanical properties in flexure loading mode generally for viscoelastic or polymer-based systems. Rectangular bars cut from molded plates were realized with nominal dimension of 2 × 10 × 60 mm. The mixture was taken from the match used by the surgeon personnel during a real operation event to account for the induced variability of the “on-site” handling and the specific medical operator.

In this work, a DMA Q800 by TA INSTR set with a cooling unit was employed to perform the experiments. According to the test, a rectangular cross-section bar is placed on the DMA supports and loaded to achieve a constant elastic displacement (i.e., 40 μm) at 1 Hz frequency and 1 °C/min heating ramp. The considered span-to-thickness ratio induces a minimization of the shear forces during the flexural deformation and thus the final modulus can be assumed as the tensile modulus with a very good approximation.

**Figure 9:**
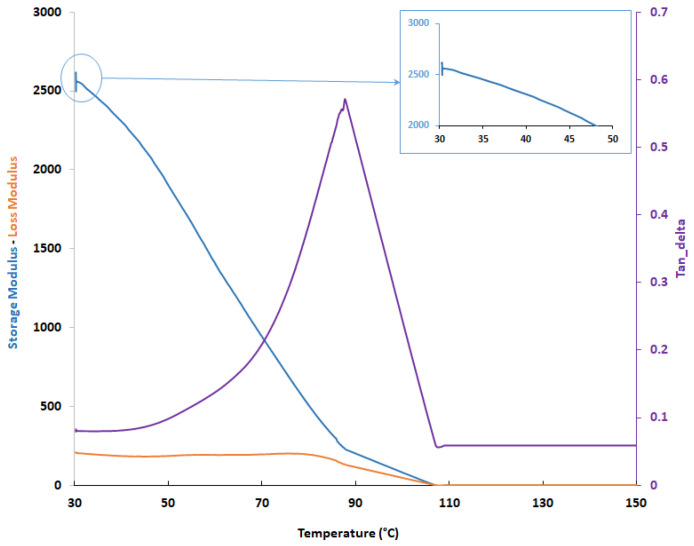
Material property variation in extended temperature range detailed.

Results indicate a sharp variation of the material stiffness sooner above 40 °C with a wide glass transition range up to 90 °C, above this temperature the sample completely deforms reporting a negligible stiffness un the considered displacement. The curve for one of the tested samples is shown in Figure 9.

Within the range 34 °C to 41 °C, the bone cement reports an expected linear dependency with the temperature, but this effect is assumed negligible within the range of medical and diagnosis interest (Figure 9).

### 3.3. Thermo-Gravimetrical Analysis

**Figure 10:**
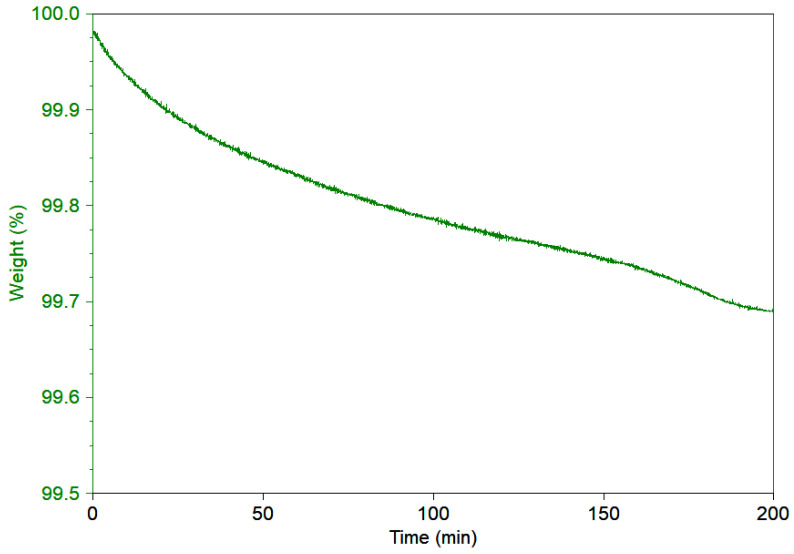
Weight loss reduction in air at isothermal temperatures.

Bone cement weight loss as a function of time, at a constant temperature (corresponding to the internal temperature of the human body of 37°) was followed by gravimetrical analysis. The test was performed on few milligrams of antibiotic bone cement taken from different location of the casted sample and heated at 1 °C/min under air for 200 min, corresponding to a time period equivalent to a surgery time frame (see Figure 10).

The weight variation is very negligible, less than 3%, validating the assumption that during the progression of the surgery the diffusion depletion of the mixed antibiotic content is unexpected, and the material will be triggering the drug release by such factors as pH blood and body location humidity. The initial drop of the curve is reasonably related to the surface absorption of antibiotic al-most immediately lost by the sample.

### 3.4. Evaluation of the Coefficient of Thermal Expansion

**Figure 11:**
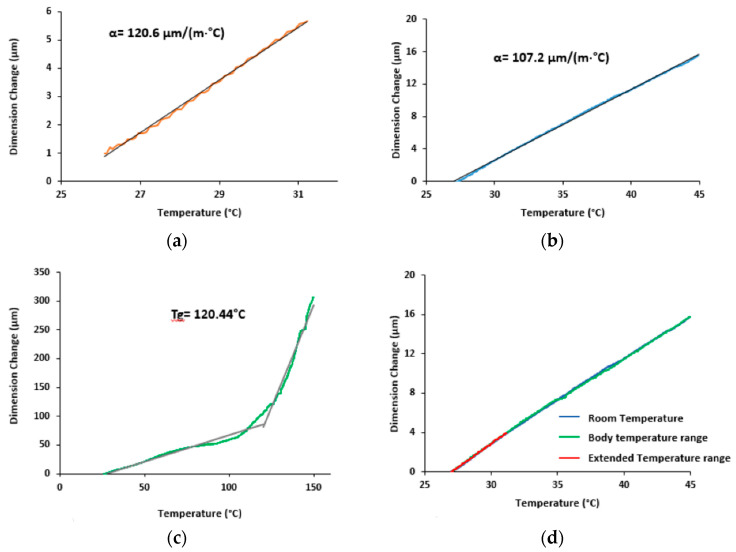
The 10 TMA curves, respectively, (**a**) at room, (**b**) body and (**c**) extended temperature range along with (**d**) overlay of the curves below the glass transition temperature.

The analysis of the thermomechanical behavior of the cement spacer was performed considering different temperature range (Figure 11), respectively, (a) ambient conditions (i.e., 26–31 °C), (b) body temperature range (i.e., 27–42 °C), (c) extended range (i.e., 21–150 °C). For each monitored curves the coefficient of thermal expansion was computed and reported along with the glass transition temperature for the extended range.

The TMA results highlight theta the coefficient of thermal expansion is within a narrow range of variation between the room condition and the higher body range as also confirmed by the test per-formed within the extended temperature range and the coefficient value reach an averaged value of 118.9 °mm/°C.

### 3.5. Simulation Data Analysis and Discussion

**Figure 12:**
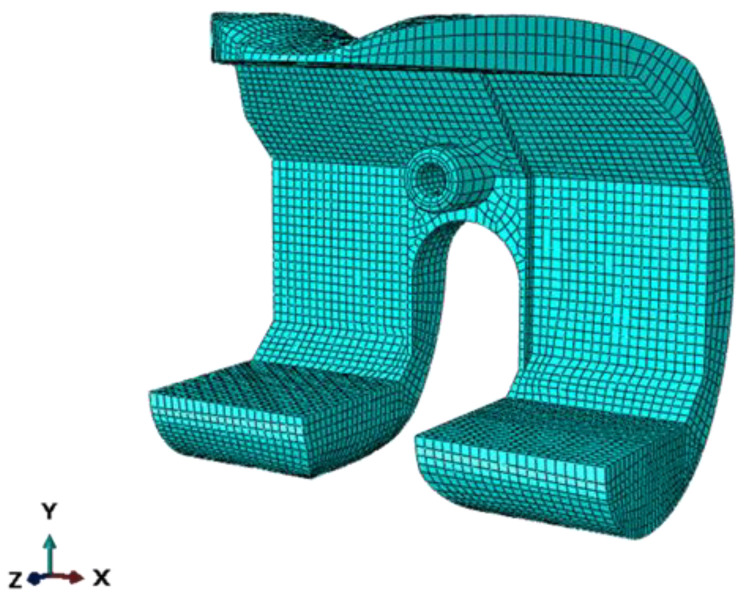
Finite Element Model of the joint replacement.

In the framework of optimized and customizable design, the use of 3D printing process [29,30] is mandatory to produce the joint replacement. This kind of manufacturing technique, however, requires a series of preliminary structural analyses to verify the resistance of the products when subjected to service loading conditions [31]. Hence, to verify the joint replacement, numerical analyses have been carried out, by using the mechanical properties of the antibiotic cement derived from the experimental tests previously described. The Finite Element Analyses have been carried out in the ABAQUS environment. The structure has been discretized by using solid elements with a reduced integration scheme (C3D8R). The Finite Element Model of the joint replacement is shown in Figure 12 [32].

**Figure 13:**
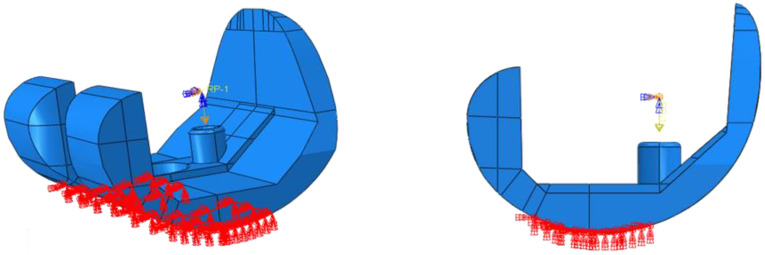
Test Case 1: Boundary conditions.

**Figure 14:**
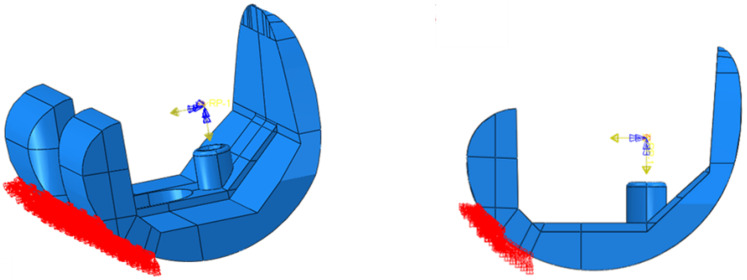
Test Case 2: Boundary conditions.

**Figure 15:**
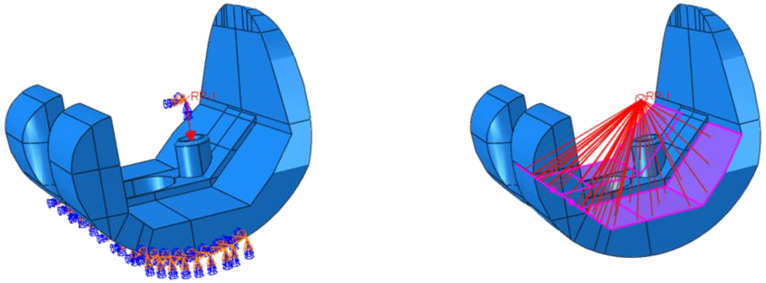
Test Case 1: Applied load and coupling constraints.

**Figure 16:**
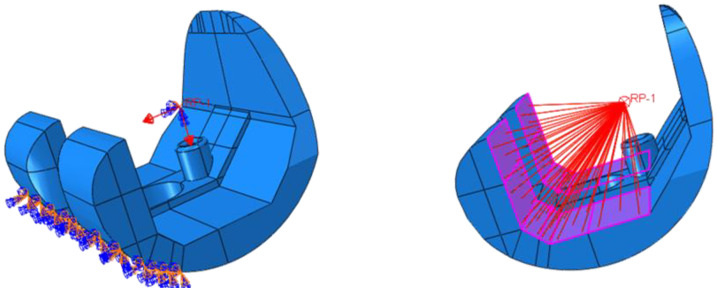
Test Case 2: Applied load and coupling constraints.

Two test cases have been considered: level walking (Test Case 1) and 45° bent knee (Test Case 2). The boundary conditions used to simulate the level walking and the 45° bent knee test cases are shown in Figure 13 and Figure 14, respectively. According to these figures, only the surfaces resting on the tibial component have been constrained. To highlight this aspect, Figure 15 and Figure 16 report photos of the real component in Test Case 1 and Test Case 2 configuration.

Moreover, a 2000 N load, equivalent to the load acting on the knee by a 70 kg walking man (multi-plied by a factor of about 300%) [33] and oriented in the direction of the tibial component, has been applied to a reference point, coupled to the surfaces in contact to the thighbone, as shown in Figure 15 and Figure 16 for the Test Case 1 and Test Case 2, respectively.

Then, static non-linear analyses have been carried out on each Test Case, to determine the stress distribution on the component when subjected to the service loading conditions.

**Figure 17:**
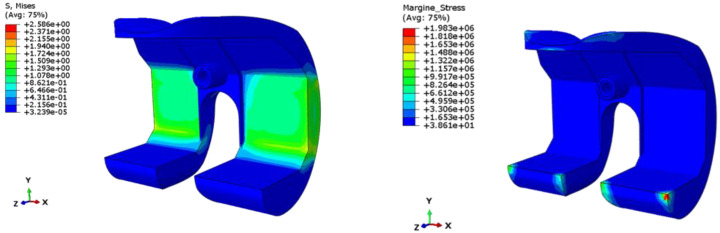
Test Case 1: Stress field and safety margins.

**Figure 18:**
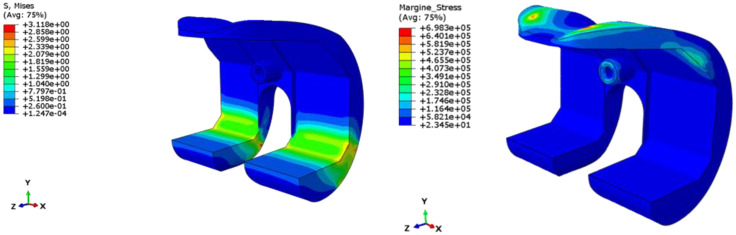
Test Case 2: Stress field and safety margins.

In Figure 17 and Figure 18, the results, in terms of von Mises stress and safety margins are reported for Test Case 1 and Test Case 2, respectively. In particular, the safety margins are representative of the stress level σ respect to the critical stress value of the material σ_crit_. Values of the safety margins lower than 0 indicates that the stress of the structure locally exceeds the critical value of the stress, while positive safety margins are representative of values of the stress lower than the critical one. Higher values of the safety margins indicate higher differences between local and critical stresses. In particular, the safety margin is computed as [34]:

(1) $$Safety\;Margins=\frac{\sigma_{crit}}{\sigma }-1$$

According to Figure 17 and Figure 18, the value of the stress for both loading conditions does not exceed the critical value of the material, which is different orders of magnitude higher than the local stresses of the structure. Hence, it can be concluded that the spacer is properly designed, and no criticalities could be experienced during the service.

## 4. Conclusions

In this work, the customization of the spacer has been assessed, based on the mechanical performance in operating and simulated worst loading conditions. The mechanical characteristics of the materials of the prototype have been derived by means of experimental test procedures, using the ASTM standards F451, D5023, and E831. Additionally, thermo-gravimetrical analyses have been carried out to evaluate the weight variation during the progression of the surgery.

The analyses have demonstrated that the weight loss is very negligible (<3%) for the considered conditions; moreover, the mechanical performances of the spacer have been investigated by means of finite element approaches in two referred loading modes. The numerical analyses suggested that the spacer is highly over-dimensioned. Actually, suitable configurations, capable of sustaining safely the service operative conditions, can be obtained by reducing, in the most critical regions, the thickness up to an order of magnitude. Indeed, future development could be focused on the size and shape optimization of the spacer, to reduce its total mass especially in the least stressed regions.

## Data Availability

The data presented in this study are available on request from the corresponding author.

## References

[1] Centers for Disease Control and Prevention (2013). National Hospital Discharge Survey: 2010 Table, Procedures by Selected Patient Characteristics.

[2] Kurtz S., Ong K., Lau E., Mowat F., Halpern M. (2007). Projections of Primary and Revision Hip and Knee Arthroplasty in the United States from 2005 to 2030. J. Bone Jt. Surg..

[3] Havelin L.I., Fenstad A.M., Salomonsson R., Mehnert F., Furnes O., Overgaard S., Pedersen A.B., Herberts P., Kärrholm J., Garellick G. (2009). The Nordic Arthroplasty Register Association: A unique collaboration between 3 national hip arthroplasty registries with 280,201 THRs. Acta Orthop..

[4] Robertsson O., Bizjajeva S., Fenstad A.M., Furnes O., Lidgren L., Mehnert F., Odgaard A., Pedersen A.B., Havelin L.I. (2010). Knee arthroplasty in Denmark, Norway and Sweden. Acta Orthop..

[5] Singh J.A., Yu S., Chen L., Cleveland J.D. (2019). Rates of Total Joint Replacement in the United States: Future Projections to 2020–2040 Using the National Inpatient Sample. J. Rheumatol..

[6] Patel R., Rodríguez-Merchán E., Oussedik S. (2015). Aseptic Failure in Total Knee Arthroplasty. Total Knee Arthroplasty.

[7] Balato G., Ascione T., Iorio P., De Franco C., De Matteo V., D’Addona A., Tammaro N., Pellegrino A. (2019). Knee septic arthritis caused by α-hemolytic Streptococcus in a patient with a recent history of knee arthroscopy: A case report. BMC Infect. Dis..

[8] Tande A.J., Patel R. (2014). Prosthetic Joint Infection. Clin. Microbiol. Rev..

[9] Garfield K., Noble S., Lenguerrand E., Whitehouse M.R., Sayers A., Reed M.R., Blom A.W. (2020). What are the inpatient and day case costs following primary total hip replacement of patients treated for prosthetic joint infection: A matched cohort study using linked data from the National Joint Registry and Hospital Episode Statistics. BMC Med..

[10] Smeraglia F., Soldati A., Orabona G., Ivone A., Balato G., Pacelli M. (2015). Trapeziometacarpal arthrodesis: Is bone union necessary for a good outcome?. J. Hand Surg. (Eur. Vol.).

[11] Bozic K.J., Ries M.D. (2005). The impact of infection after total hip arthroplasty on hospital and surgeon resource utilization. J. Bone Jt. Surg..

[12] Kurtz S.M., Lau E., Watson H., Schmier J.K., Parvizi J. (2012). Economic Burden of Periprosthetic Joint Infection in the United States. J. Arthroplast..

[13] Parisi T.J., Konopka J.F., Bedair H.S. (2017). What is the long-term economic societal effect of peripros-thetic infections after THA? A Markov analysis. Clin. Orthop. Relat. Res..

[14] Patel A., Pavlou G., Mújica-Mota R.E., Toms A.D. (2015). The epidemiology of revision total knee and hip arthroplasty in England and Wales a comparative analysis with projections for the United States. A study using the National Joint Registry dataset. Bone Jt. J..

[15] Vanhegan I.S., Malik A.K., Jayakumar P., Ul Islam S., Haddad F.S. (2012). A financial analysis of revision hip arthroplasty: The economic burden in relation to the national tariff. J. Bone Jt. Surg..

[16] Klouche S., Sariali E., Mamoudy P. (2010). Total hip arthroplasty revision due to infection: A cost analysis approach. Orthop. Traumatol. Surg. Res..

[17] Haddad F.S., Muirhead-Allwood S.K., Manktelow A.R.J., Bacarese-Hamilton I. (2000). Two-stage uncemented revision hip arthroplasty for infection. J. Bone Jt. Surg. Br. Vol..

[18] Hsieh P.-H., Shih C.-H., Chang Y.-H., Lee M.S., Yang W.-E., Shih H.-N. (2005). Treatment of deep infection of the hip associated with massive bone loss: Two-stage revision with an antibiotic-loaded interim cement prosthesis followed by reconstruction with allograft. J. Bone Jt. Surg. Br. Vol..

[19] Citak M., Argenson J.N., Masri B., Kendoff D., Springer B., Alt V., Taunton M.J., Vogely C.H., Wellman S.S. (2014). Spacers. J. Arthroplast..

[20] Balato G., Ascione T., Rosa D., Pagliano P., Solarino G., Moretti B., Mariconda M. (2015). Release of gentamicin from cement spacers in two-stage procedures for hip and knee prosthetic infection: An in vivo pharmacokinetic study with clinical follow-up. J. Biol. Regul. Homeost. Agents.

[21] Balato G., Roscetto E., Vollaro A., Galasso O., Gasparini G., Ascione T., Catania M.R., Mariconda M. (2019). Bacterial biofilm formation is variably inhibited by different formulations of antibiotic-loaded bone cement in vitro. Knee Surg. Sports Traumatol. Arthrosc..

[22] Pascarella R., Cerbasi S., Politano R., Balato G., Fantasia R., Orabona G., Mariconda M. (2017). Surgical results and factors influencing outcome in patients with posterior wall acetabular fracture. Injury.

[23] Van Thiel G.S., Berend K.R., Klein G.R., Gordon A.C., Lombardi A.V., Della Valle C.J. (2011). Intraoperative Molds to Create an Articulating Spacer for the Infected Knee Arthroplasty. Clin. Orthop. Relat. Res..

[24] Chang Y., Lee M.S., Liau J.-J., Liu Y.-L., Chen W.-C., Ueng S.W.N. (2020). Polyethylene-Based Knee Spacer for Infection Control: Design Concept and Pre-Clinical In Vitro Validations. Polymers.

[25] Amanatullah D., Dennis D., Oltra E.G., Gomes L.S.M., Goodman S.B., Hamlin B., Hansen E., Hashemi-Nejad A., Holst D.C., Komnos G. (2019). Hip and Knee Section, Diagnosis, Definitions: Proceedings of International Consensus on Orthopedic Infections. J. Arthroplast..

[26] Balato G., Ascione T., De Franco C., De Matteo V., Verrazzo R., Smeraglia F., Rizzo M., Bernasconi A., Mariconda M. (2020). Blood loss and transfusion rate in patients undergoing two-stage exchange in infected total knee arthroplasty. J. Biol. Regul. Homeost Agents.

[27] Balato M., Petrarca C., de Matteo V., Lenzi M., Festa E., Sellitto A., Campi J., Zarrelli M., Balato G. (2021). On the Necessity of a Customized Knee Spacer in Peri-Prosthetic Joint Infection Treatment: 3D Numerical Simulation Results. J. Pers. Med..

[28] https://www.zimmerbiomet.com/content/dam/zb-corporate/en/products/specialties/cement/refobacin-bone-cement-r/16361USenBiometBCRandRefobacinBCRProductSheetdigital.pdf.

[29] Smith M. (2019). ABAQUS/Explicit User’s Manual.

[30] Berman B. (2012). 3-D printing: The new industrial revolution. Bus. Horiz..

[31] Tofail S.A.M., Koumoulos E.P., Bandyopadhyay A., Bose S., O’Donoghue L., Charitidis C. (2018). Additive manufacturing: Scientific and technological challenges, market uptake and opportunities. Mater. Today.

[32] Di Caprio F., Acanfora V., Franchitti S., Sellitto A., Riccio A. (2019). Hybrid Metal/Composite Lattice Structures: Design for Additive Manufacturing. Aerospace.

[33] Otten E. (2003). Inverse and forward dynamics: Models of multi–body systems. Philos. Trans. R. Soc. B Biol. Sci..

[34] Ramsey J.K. (2019). Calculating Factors of Safety and Margins of Safety from Interaction Equations, NASA Scientific and Technical Information (STI). Program. https://ntrs.nasa.gov/api/citations/20190032150/downloads/20190032150.pdf.

[35] Rasmussen L.S., Gisvold S.E., Wisborg T. (2014). Ethics Committee approval for observational studies. Acta Anaesthesiol. Scand..

